# Long-Term Neuropsychological Outcome in Preterm Twins

**DOI:** 10.1100/tsw.2006.175

**Published:** 2006-08-04

**Authors:** Giovanni Iannone, Clelia Tripaldi, Antonino Chindemi, Lorenzo Piscitelli, Antonio Mastrorocco, Silvano Palazzo, Luigi Esposito

**Affiliations:** ^1^ Neonatal Intensive Care Unit, “F. Miulli” Hospital, Acquaviva delle Fonti (Ba), Italy; ^2^ Paediatric Hospital “Giovanni XXIII”, University of Bari, Italy; ^3^ Child Neuropsychiatry Unit, “F. Miulli” Hospital, Acquaviva delle Fonti (Ba), Italy; ^4^ Department of Emergency and Organs Transplantation, University of Bari, Italy

**Keywords:** preterm twins, neuropsychological outcome, self-regulatory processes

## Abstract

Few long-term studies have yet described neuropsychological outcome in preterm twins. Our aim was to assess, by long-term evaluation, neuropsychological outcome in preterm twins in order to define a correct follow-up program. Our study was a cohort one, with an index and a comparison group. Neonatal medical records of all preterm newborns admitted to our centre between 1991 and 1997 were reviewed and selected patients were recalled. The sample population included two matched groups of children aged 6—12 years, 86 twins and 86 singletons, submitted to paediatric, neurological, psychological, and ophthalmological examinations. Inclusion criteria were twin pregnancy and gestational age 27—36 weeks for index group; same gestational age, but single pregnancy, for the comparison group. All children underwent paediatric and neuropsychiatric examinations, cognitive assessment, and psychological evaluation by standardized tests for screening of learning specific disorders and language difficulties, and finally, ophthalmological examination. In order to study their role in predicting neuropsychological outcome, we examined some perinatal prognostic factors by statistical analysis. Unfavourable neuropsychological outcome was observed in 55/172 (32%) children, with different prevalence in the two groups, 42/172 (24%) in twins and 13/172 (8%) in singletons. Statistical analysis performed for examined prognostic factors showed significant differences in neuropsychological outcome with regard only to gestational age < 32 weeks, low birth weight, intraventricular haemorrhage, and periventricular leukomalacia. The incidence of neuropsychological diseases in the two groups showed significant difference about language and learning difficulties. Our data suggest that preterm twins represent a particular high-risk category of premature babies, mostly regarding the risk of so-called “minimal brain dysfunction”, so a careful follow-up is recommended.

## INTRODUCTION

The progress in neonatal medical care has allowed survival of very low birth-weight children. Despite the important improvement of survival and outcome, a great number of neuropsychological long-term sequelae have been reported[1], so that it is very important to organize articulated follow-up programs. In the last 15 years, because of the increase in assisted conception, the incidence of multiple pregnancies has grown rapidly. These pregnancies are burdened with obstetric and neonatal complications, often associated, which may cause intrauterine growth retardation and premature birth. Twin babies are a group of preterm newborns at high risk of an unfavourable long-term neuropsychological outcome.

Few studies have yet described the long-term neuropsychological outcome in preterm twins, regardless of the zygosity issue and the cotwin survival rate. Short-term follow-up studies may precisely identify the more severe sequelae, but only long-term evaluations can diagnose minimal brain dysfunction.

Low birth-weight, critical, preterm newborns represent the first category at risk for brain damage. Retrospective studies[1] have shown that ELBW newborns have the worst neurological outcome. Moreover, they have a high incidence of behavioural disorders and attention defects, while VLBW newborns present a greater incidence of cognitive sectorial disturbances, especially regarding nonverbal abilities, memory, fine motility, and visual-motor integration. These cognitive impairments do not become manifest before 5–7 years.

According to Pharoah[2], the incidence of cerebral palsy in term twins is 5–10 times higher than in singletons of the same gestational age whereas, for low gestational ages, there is the same incidence in the two groups of babies. In a prospective study from 1998[3] regarding learning disorders in 28 school-aged twin pairs, it was shown that the commonest disturbances were writing and reading difficulties. In fact, 12.5% of twins had dyslexia and 10.7% dysgraphia, while about 20% had poor visual-motor coordination.

Regarding the etiopathogenesis of neurological damage in twins, there are several likely explanations. In monochorial twins with precocious intrauterine twin death, the passage of thromboplastin material by vascular anastomosis may lead to brain injury in the survivor[4]. Some interferences in the organizational events of the brain, haemodynamic jumps, adverse effects of medical injury and neonatal stress, obstetric complications, and feto-fetal transfusion may lead to brain damage.

The purpose of our study was to identify and describe the long-term neuropsychological outcome in preterm twins, in order to define a correct follow-up program for these babies.

## MATERIALS AND METHODS

This study was a cohort one, 3-years lasting, with an index and a comparison group. Neonatal medical records of all preterm twin and preterm singleton patients admitted to our centre (“F. Miulli” Hospital, Acquaviva delle Fonti, Bari, Italy) between 1991 and 1997 were reviewed. The selected twins were all preterm twins born between 1991 and 1997 (27 weeks < GA < 36 weeks). In order to evaluate the results, we matched two groups of preterms: singletons and twins. Our data were obtained from a population of 172 children, aged 6–12 years, 86 twins and 86 singletons, all ex-preterm (Table 1). The 86 twins came from 43 twin pregnancies. The 86 selected singletons were randomized from a sampling frame of 1060 children born between 1991 and 1997. Inclusion criteria were only gestational age 27–36 weeks and single pregnancy. The two groups were matched for sex, current age, and gestational age. The twins group included 38 monochorial and 48 bichorial twins. Two patients with genetic or chromosomal disorders were excluded.

Variances regarding the starting features of children belonging to twin and singleton populations have been compared by Fisher test (equality of variances test), which did not find any statistical significant difference between the two groups (Table 2).

All the children, followed up by outpatient controls until the age of 3, were recalled.

We organized an examination program, according to the guidelines of the Italian Society of Childhood and Adolescence Neuropsychiatry[5], including: anamnesis, general examination, neurological examination, psychodiagnostic assessment, clinical observation and parents' interview, and finally, ophthalmological examination.

**TABLE 1**
**Features of Selected Patients at Birth**

| | Twins | Singletons |
|---|---|---|
| Male | 43 | 44 |
| Female | 43 | 42 |
| Range EG (wks) | 27-36 | 27-36 |
| Weight range (g) | 780-2530 | 725-2495 |
| G.A. <32 wks | 60 | 60 |
| LBW | 47 | 50 |
| VLBW | 33 | 29 |
| ELBW | 6 | 7 |
| SGA | 6 | 8 |
| Caesarean deliveries * | 53 | 61 |
| Born from assisted conception * | 7 | 1 |

* $p < 0.05$

**TABLE 2**
**Analysis of Variances Regarding the Starting Features of Patients of the Twin Group and of Singleton Group**

| Parameters | Variance in twin group | Variance in singleton group | p |
|---|---|---|---|
| Apgar score | ± 2.9 | ± 2.7 | 0.7 |
| Gestational age | ± 4.35 | ± 4.48 | 0.89 |
| Birth-weight | ± 1622.87 | ± 1473.29 | 0.66 |

We organized an examination program, according to the guidelines of the Italian Society of Childhood and Adolescence Neuropsychiatry[5], including: anamnesis, general examination, neurological examination, psychodiagnostic assessment, clinical observation and parents' interview, and finally, ophthalmological examination.

Anamnesis regarded child’s developmental assessment of motor, linguistic, reading, writing, calculating and praxis abilities, relational and affective organization, home environment, and presence of neuropsychiatric diseases (seizures, headaches, movement disorders, sleep disorders, behavioural disorders).

General examination regarded assessment of growth (weight, length, head circumference). Neurologic evaluation considered posture, motor function, fine motor organization, praxia, visual motor coordination, lateral prevalence, child’s behaviour and degree of cooperation, pathological signs.

Psychodiagnostic evaluation included cognitive assessment by the Wechsler Intelligence Scale for Children (WISC)[6]. Those obtaining an unsatisfactory score at the WISC (total score < 85) underwent Tzuriel’s Test[7], a battery of items exploring deductive reasoning abilities, in order to evaluate their dynamic behavioural skill. The psychologist’s work included the administration of a language assessment test for a screening evaluation of expressive and receptive language, Cianchetti and Sannio’s Test[8].

Furthermore, every child was submitted to an individual screening, standardized level test in order to examine his/her specific abilities in reading and writing; and Cornoldi’s battery of tests for evaluation of subcomponents of reading and dictation. This set of tests includes 12 items, 9 for reading analysis in the components of correct and fast reading and text comprehension, and 3 for orthographic dictation. Obtained results have been analysed by a final score given from the total number of mistakes, and a deviation score was computed to a diagram for age and class. Those obtaining a score < 2DS under the average were sent to territorial psychology services for further evaluations[9]. Children attending the first elementary class were recalled at the end of the first school year to avoid evaluation errors.

The neuropsychological examination included the diagnosis of disorders of self-regulatory processes, representing a new group of emergent psychological impairments[10]. The diagnosis of disorders of self-regulatory processes, which consider the emotional, behavioural, and motor organization of the child and his/her ability in processing outer information, was assessed only by clinical observation according to clinical criteria of 0–3 Diagnostical Classification[11].

The psychologist encouraged free talk in order to evaluate the emotional and relational organization of the child. Finally, all the children underwent clinical examination from an ophthalmologist for assessment of eye movement, alignment, and visual impairment.

In order to study any roles in predicting neuropsychological outcome, we examined other perinatal prognostic factors taken from clinical diary such as gestational age, 5-min Apgar score, birth-weight categories, sex, caesarean birth, mono/bichoriality, induced pregnancy, intrauterine growth retardation, severe asphyxia, hyaline membrane disease, intraventricular haemorrhage, periventricular leukomalacia, jaundice, and sepsis. Statistical analysis was performed by Kaplan-Meier survival plots to evaluate the neuropsychological outcome in the sample in layers for prognostic factors. The degree of significance established for all the statistical tests was $p < 0.05$. We also used a multivariate statistical analysis by Cox’s model to evaluate the roles of each prognostic factor on the final neuropsychological outcome.

## RESULTS

An unfavourable neuropsychological outcome was observed in 55/172 (32%) children, with a different prevalence in the two groups, 42/172 (24%) in twins and 13/172 (8%) in singletons ($p = 0.0048$). The different clinical diseases observed in the two groups are shown in Table 3.

Epilepsy was observed in 5/86 (5.8%) of twins vs. 1/86 (1.1%) of singletons ($p = 0.09$). Cerebral palsy had the same incidence, being 8/86 (9.3%) in the two groups ($p = 0.9$).

Mental retardation, (IQ < 70) was observed in 2/86 (3.3%) of twins vs. 1/86 (1.1%) of singletons ($p = 0.5$).

Language-specific disturbances were present in 7/86 (8.1%) of twins vs. 1/86 (1.1%) of singletons ($p = 0.03$). Five of these children had an inadequate score in verbal tests.

Specific learning difficulties (dyslexia, dysgraphia) were present in 6/86 (6.9%) of twins vs. 0/86 (0%) of singletons ($p = 0.01$). All these children had an inadequate score in performance tests, so they underwent Tzuriel’s test for dynamic assessment, with good results, and were then sent to language rehabilitation services.

Disorders in self-regulatory processes had an incidence of 6/86 (6.9%) in twins vs. 2/86 (3.3%) in singletons ($p = 0.14$).

Kaplan-Meier analysis performed for the other examined prognostic factors showed significant differences in neuropsychological outcome with regard only to gestational age < 32 weeks ($p = 0.0247$), low birth weight ($p = 0.0001$), intraventricular haemorrhage ($p = 0.037$), and periventricular leukomalacia ($p = 0.0001$), as shown in Fig. 1. Multivariate analysis confirmed the data obtained with the Kaplan-Meier plots, showing that those parameters with statistical value confirmed by univariate analysis kept their predictive value also in the Cox-model, independently influencing the prognosis. It was found that the risk for later neuropsychological disease was 3.2 times greater in twins than in singletons ($p = 0.0026$, I.C: 1.50–6.89) (see Table 4).

**TABLE 3**
**Neuropsychological Outcome Observed in the Examined Groups**

| Disease | N. patients | % | N. twins | %twins | N. singletons | % singletons | p |
|---|---|---|---|---|---|---|---|
| Epilepsy | 6 | 3.5 | 5 | 5.8 | 1 | 1.1 | 0.09 |
| Cerebral palsy | 16 | 9.3 | 8 | 9.3 | 8 | 9.3 | 0.9 |
| Mental retardation | 3 | 1.7 | 2 | 3.3 | 1 | 1.1 | 0.5 |
| Troubles of Self-regulation processes | 8 | 4.6 | 6 | 6.9 | 2 | 3.3 | 0.14 |
| Learning difficulties ** | 6 | 3.5 | 6 | 6.9 | 0 | 0 | 0.01 |
| Language disorders ** | 8 | 4.6 | 7 | 8.1 | 1 | 1.1 | 0.03 |

** Underlines statistic-significant differences.

Kaplan-Meier analysis performed for the other examined prognostic factors showed significant differences in neuropsychological outcome with regard only to gestational age < 32 weeks ($p = 0.0247$), low birth weight ($p = 0.0001$), intraventricular haemorrhage ($p = 0.037$), and periventricular leukomalacia ($p = 0.0001$), as shown in Fig. 1. Multivariate analysis confirmed the data obtained with the Kaplan-Meier plots, showing that those parameters with statistical value confirmed by univariate analysis kept their predictive value also in the Cox-model, independently influencing the prognosis. It was found that the risk for later neuropsychological disease was 3.2 times greater in twins than in singletons ($p = 0.0026$, I.C: 1.50–6.89) (see Table 4).

The ophthalmological examination showed that 30/86 (34.8%) of twins and 15/86 (17.4%) of singletons had visual impairment (myopia, astigmatism, hypermetropia), and so twin birth resulted to be an important prognostic factor also for visual function ($p$ =0 .01).

Kaplan-Meier analysis of neurological sequelae in patients born from assisted conception shows no statistical significant difference towards those conceived naturally ($p = 0.45$).

## DISCUSSION

Our study partly confirms the data in literature regarding the increased incidence of neuropsychological diseases in twins and in preterm newborns. In our study, we compared two groups of preterm newborns, twins and singletons. Our results showed a strong influence of twin birth on neuropsychic outcome, while according to previous studies[12], this parameter should be influenced only by an increased risk of preterm delivery. The more severe neurological diseases, including epilepsy, cerebral palsy, and mental retardation, are clearly influenced by prematurity and low birth weight.

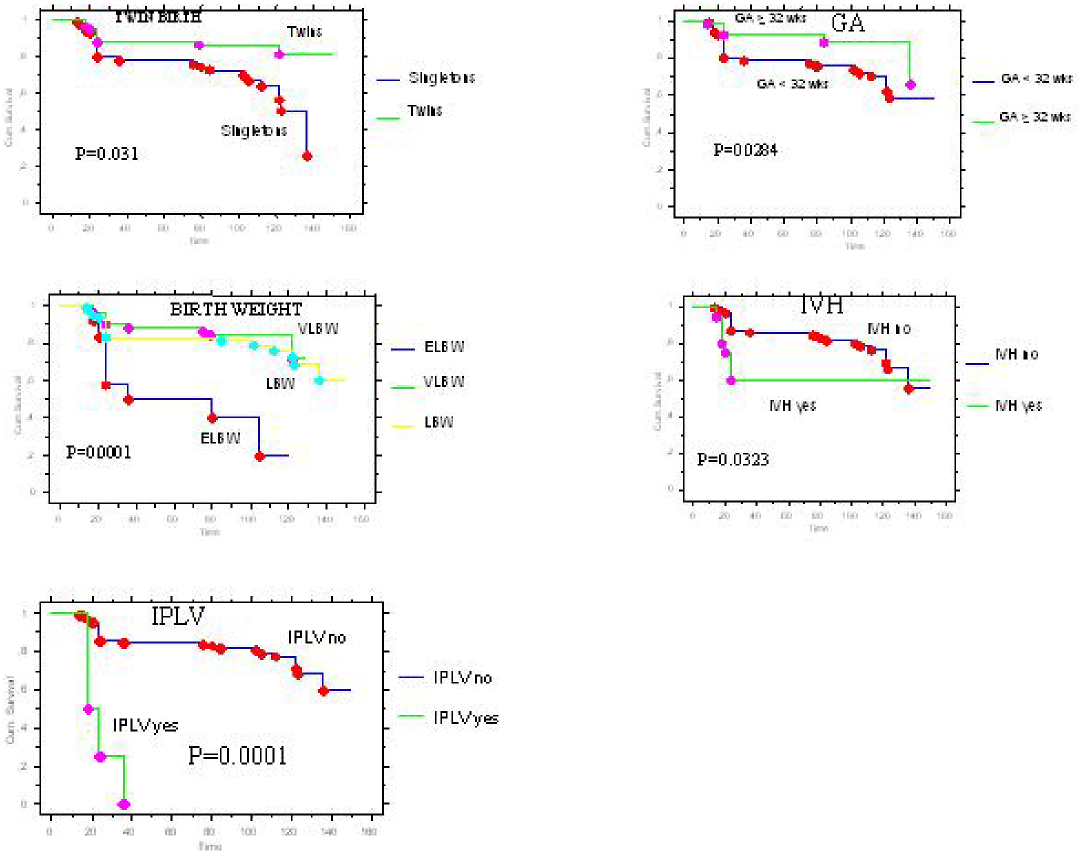

**FIGURE 1.** Kaplan-Meier univariate analysis for twinning, gestational age (GA) groups, weight range, intraventricular haemorrhage (IVH, periventricular leukomalacia (IPLV).

According to Pharoah, the incidence of cerebral palsy in term twins is 5–10 times higher than in singletons of the same gestational age[2], whereas there is no difference in this incidence in the two groups for low gestational ages. Our data, in agreement with Pharoah's, showed no differences regarding more severe neurological diseases between the two groups of children examined; this also confirms the results of several studies by Nelson[13,14]. According to Pharoah, the likely pathogenesis of neurological damage should be haemodynamic imbalances due to placental vascular anastomoses[15] and neurological impairment should, therefore, be more frequent in monochorionic twins. Our data do not confirm any statistically significant difference in neuropsychic outcome between monochorial and bichorial twins ($p$ = 0.57).

With regard to epilepsy, the 3% incidence we found in the preterm twins group is near to the data of Scher et al.[16], reporting an incidence of 4.7% in twins. Regarding mental retardation, we found an incidence of 1.2% in preterm twins, about double that in singletons; this agrees with Broman's data, which describe a threefold incidence in twins, including those at full-term[17].

In our experience, the most important developmental disturbances in twins are language difficulties, especially at the beginning of primary school. The case histories of Haverkamp et al. showed an incidence of language disturbance in 13% of 94 twins[18], while our data, on a smaller sample, showed an incidence of 8.7%. An important phenomenon that could influence the language difficulties is the use of a secret language in the twin couple, already described[3]. The presence of secret language seems to be a developmental phenomenon occurring in the second year of life, with the emergence of immature speech.

**TABLE 4**
**Cox-Model Multivariate Statistical Analysis for Study of Prognostic Factors.**

| Variable | p-value | Exp (Coef) | 95%lower | 95% Upper |
|---|---|---|---|---|
| **Twin birth** ** | 0.0026 | 3.221 | 1.505 | 6.891 |
| **Apgar score (5')** | 0.7273 | 0.835 | 0.303 | 2.300 |
| GA * | 0.0414 | 2.776 | 1.040 | 7.406 |
| **Birth weight** ** | 0.0124 | - | - | - |
| ELBW vs LBW | 0.0628 | 2.824 | 0.946 | 8.429 |
| VLBW vs LBW | 0.1506 | 0.555 | 0.248 | 1.239 |
| **SGA** | 0.8060 | 1.185 | 0.305 | 4.599 |
| **Asphyxia** | 0.7482 | 1.126 | 0.545 | 2.328 |
| **HMD** | 0.5132 | 0.783 | 0.377 | 1.629 |
| **IPLV** | 0.1106 | 0.360 | 0.102 | 1.263 |
| **IVH** ** | 0.0049 | 0.273 | 0.110 | 0.675 |

*Note:* * and ** underline, respectively, statistic-significant and very significant differences. GA, gestational age; ELBW, extremely low birth weight; VLBW, very low birth weight; LBW, low birth weight; SGA, small for gestational age; HMD, hyaline membranes disease; IPLV, periventricular leukomalacia; IVH, intraventricular haemorrhage.

The studied group of children was reported to use a private language at 36 months. This group had poorer cognitive and language functioning, and was characterized by highly dependent relationships. The follow-up of these children at the age of 6 showed that language outcome was poor for the subgroup who did not develop normal language alongside the use of a private language[21]. The relation of secret language with articulation delays is actually discussed[22]. It could probably interfere with the correct learning of words, and later with writing and reading abilities, but it cannot be considered a causal factor of speech delay, only a risk factor. In our experience, a secret language was observed in 40.6% of twins, while only 13% of the twins had language delays. The use of a secret language may cause twin children to experience learning and writing difficulties. These disturbances are rarely diagnosed before school age because, especially during the first years of life, they are explained as variants of normal development.

From our data, it emerges that intrauterine growth retardation, despite not being a significant variable in long-term neuropsychological outcome ($p$ = 0.078), may have a borderline effect on prognosis, confirming the data in literature[19]. Our work enhances the current literature data by providing information about disorders in self-regulatory processes, which were found to have a similar incidence in the two groups of children examined ($p$ = 0.14) and whose pathogenesis is due to nonmaturation of the regulation systems developing in the same period as language skills.

Numerous studies have reported an increased risk of refractive errors in prematurely born infants. Prematurity per se is associated with refractive errors. Preterm infants remain more myopic than the full-term infants[23]. Preterm infants without Retinopathy of Prematurity(ROP) have high rates of significant refractive errors. The ophthalmological examination underlines that prematurity is an important risk factor for visual impairment.

In the last years, there have been conflicting opinions about influence of assisted conception on infant neurological outcome. According to Stromberg et al.[24], children born after *in vitro* fertilization have an increased risk of developing neurological problems, especially cerebral palsy. Our evidence from analysis of the prognostic fertility factor is that twins from assisted conception have a similar risk of neurological sequelae as their naturally conceived peers. Our data agree with those of Pinborg et al.[25] who compared, in a cohort study performed on a champion of 3393 twins and 5130 singletons born from assisted conception and 10239 naturally conceived twins, incidence of neurological sequelae, which are similar in the three groups.

## CONCLUSIONS

Our data suggest that preterm twins represent a particular high-risk category of premature babies, mostly regarding the risk of so-called "minimal brain dysfunction". A careful follow-up is recommended in preterm twins. We recommend, after the reported findings, a timeline of follow-up visits. In children aged 3–8 years, it should be useful to organize periodic controls of physical and neuropsychological development and to schedule parent interviews, to evaluate their children's learning, writing, and reading skills. Until 18 months of life, we suggest 3-monthly clinical controls, then 6-monthly until the age of 3 and finally, yearly examinations until school age. Hearing screening by OAE (otoacoustic emission)[20] is recommended within the 6 month of life. Ophthalmologic assessment should be performed yearly until school age; psychological evaluation to assess cognitive skills should be performed at least once after the beginning of primary school.

This organizational model may enable early diagnosis of learning and language disturbances, and of visual and hearing complaints. Different specialists are responsible for this organization; firstly, the neonatologist and the paediatrician, and then the neuropsychiatrist, the ophthalmologist, and the psychologist because important sequelae may influence the child's overall physical and mental development. We underline the important role played by early diagnosis in order to plan adequate clinical assistance.
